# RNA-Dependent Intergenerational Inheritance of Enhanced Synaptic Plasticity after Environmental Enrichment

**DOI:** 10.1016/j.celrep.2018.03.059

**Published:** 2018-04-10

**Authors:** Eva Benito, Cemil Kerimoglu, Binu Ramachandran, Tonatiuh Pena-Centeno, Gaurav Jain, Roman Manuel Stilling, Md Rezaul Islam, Vincenzo Capece, Qihui Zhou, Dieter Edbauer, Camin Dean, André Fischer

**Affiliations:** 1 Department for Epigenetics and Systems Medicine in Neurodegenerative Diseases, German Center for Neurodegenerative Diseases (DZNE) Göttingen, von Siebold Strasse 3A, 37075 Göttingen, Germany; 2 Trans-synaptic Signaling Group, European Neuroscience Institute, Grisebachstrasse 5, 37077 Göttingen, Germany; 3 Bioinformatics Unit, German Center for Neurodegenerative Diseases (DZNE) Göttingen, von Siebold Strasse 3A, 37075 Göttingen, Germany; 4 German Center for Neurodegenerative Diseases (DZNE), Munich, Feodor Lynen Strasse 17, 81377 Munich, Germany; 5 Department of Psychiatry and Psychotherapy, University Medical Center Göttingen, Von Siebold Strasse 5, 37075 Göttingen, Germany

**Keywords:** epigenetics, brain, microRNA, memory, intergenerational, transgenerational, exercise, environmental enrichment, cognition

## Abstract

Physical exercise in combination with cognitive training is known to enhance synaptic plasticity, learning, and memory and lower the risk for various complex diseases including Alzheimer’s disease. Here, we show that exposure of adult male mice to an environmental enrichment paradigm leads to enhancement of synaptic plasticity and cognition also in the next generation. We show that this effect is mediated through sperm RNA and especially miRs 212/132. In conclusion, our study reports intergenerational inheritance of an acquired cognitive benefit and points to specific miRs as candidates mechanistically involved in this type of transmission.

## Highlights

• Exercising male mice pass a cognitive benefit to their offspring
• This phenomenon is mediated by altered expression of sperm RNA
• Levels of miR212/132 in sperm play a key role in this intergenerational effect

## Graphical Abstract

**Figure:**
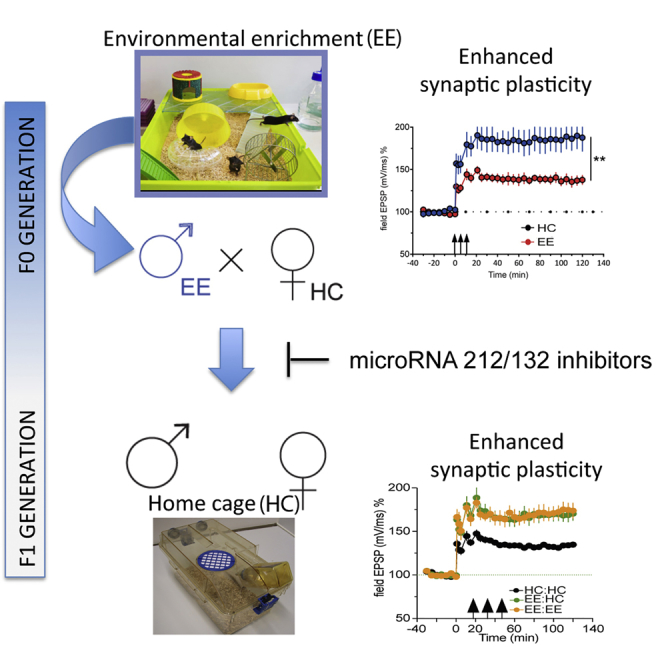

## Teaser

Environmental enrichment (EE), a combination of physical and mental exercise, has been shown to increase cognitive abilities in mice and in humans. Benito et al. find that offspring of male mice subjected to EE also show this increase. This effect is dependent on sperm RNA and involves microRNA212/132.

## Introduction

There is emerging evidence that exposure to environmental stimuli can initiate processes that transmit information to the next generation via non-genetic mechanisms (Bale, 2015, Bohacek and Mansuy, 2015, Fischer, 2014). Such forms of inter- or transgenerational inheritance have been described for aversive stimuli, such as chronic or early life stress that lead to altered response of the hypothalamic-pituitary-adrenal axis, increased anxiety and depressive-like behavior in the following generations (Gapp et al., 2014, Gapp et al., 2016, Franklin et al., 2010, Rodgers et al., 2013). There is also evidence that exposure of individuals to detrimental environmental stimuli can lead to cellular adaptations that protect the offspring when they are exposed to the same environmental insult (Zeybel et al., 2012). The idea that environmental factors can affect germ cells and thereby alter biological processes in the offspring is fascinating and may play an important role in the pathogenesis of complex diseases, especially in neuropsychiatric disorders (Bale et al., 2010, Klengel et al., 2016).

An environmental factor that was shown to lower the risk for various complex diseases, including those affecting the brain, is the combination of physical exercise and cognitive training, also called environmental enrichment (EE). EE is known to enhance synaptic plasticity in rodents and humans and is thus considered a suitable strategy to reduce the risk for dementia and other cognitive diseases (Nithianantharajah and Hannan, 2006, Fischer et al., 2007, Brown et al., 2013, Fischer, 2016). Importantly, there is evidence that exposure of juvenile mice to EE can enhance hippocampal synaptic plasticity in their offspring (Arai et al., 2009). Whether EE training in adulthood might also affect synaptic function of the next generation has not been tested so far, and the underlying mechanisms of transgenerational transmission are still poorly understood. There is, however, evidence that RNA in gametes could play a role (Gapp et al., 2014, Bale, 2015, Bohacek and Mansuy, 2015).

In this study, we demonstrate that exposure of adult mice to EE significantly enhances hippocampal LTP and cognitive function in their offspring. We show that this phenotype is due to changes in the RNA composition in the sperm of the corresponding fathers and identify microRNAs (miR) 212/132 as one factor involved in process.

## Results

### EE Training Increases Hippocampal Synaptic Plasticity in an Intergenerational Manner

**Figure 1:**
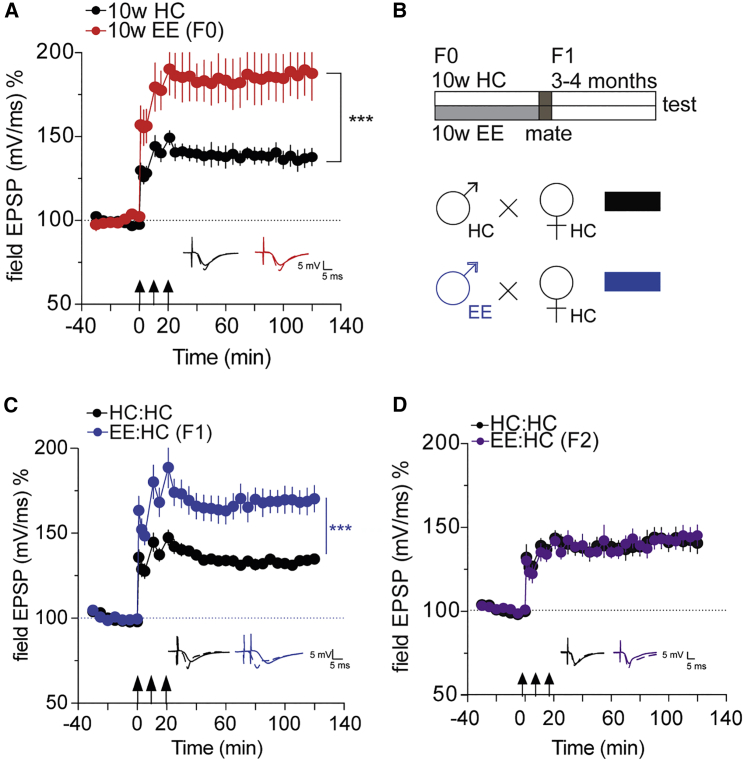
Intergenerational Inheritance of Enhanced LTP through EE (A) LTP is enhanced in 10-week EE compared to HC controls. ^∗∗∗^p < 0.001 for main effect treatment repeated-measures ANOVA (F(1,12) = 11.86); n = 8 (10w EE); 6 (10w HC). (B) Mating scheme: mice are subjected to EE for 10 weeks and mated, and the offspring are tested 3–4 months after birth. Controls spend the same amount of time in the HC. (C) LTP is enhanced in mice born to EE fathers EE:HC (n = 10) compared to HC:HC (n = 10) controls. ^∗∗∗^p < 0.001 for main effect treatment HC:HC versus EE:HC, repeated-measures ANOVA (F(1,18) = 18.74). (D) LTP is not enhanced in the F2 generation. Error bars indicate SEM.

First, we wanted to confirm that our EE protocol enhances hippocampal LTP in adult mice. To this end, we subjected mice to 10 weeks of EE training before measuring hippocampal LTP at the Schaffer Collateral CA1 synapse. We observed a highly significant increase in LTP in EE mice compared to home-caged (HC) controls (Figure 1A). Next, we wanted to test whether EE training in adult male mice would affect synaptic plasticity in their offspring (F1 generation), which are not subjected to EE. To this end, adult male mice underwent 10 weeks of EE training and were then mated to HC females (Figure 1B). We measured hippocampal LTP when the offspring were adult (3 months of age). Notably, offspring of EE fathers had increased LTP compared to those born to fathers that were housed in HC (controls; Figure 1C). The effect was similar in both male and female offspring (Figure S1). In order to account for possible confounding factors, we performed a linear regression model including the effect of paternal treatment (HC versus EE), sex (male versus female), and paternal treatment X sex interaction. In line with our previous analysis, we observed a highly significant effect of paternal treatment (p < 0.0001). We then tested whether this phenotype is passed on to the grandoffspring of the original EE animals, representing the F2 generation. Here, we did not observe any difference in the LTP between the groups (Figure 1D). These data indicate that EE in male mice leads to enhanced hippocampal synaptic plasticity in offspring, but that this effect represents inter- (and not trans-) generational inheritance.

### Intergenerational Enhancement of LTP Is Mediated via Sperm RNA

**Figure 2:**
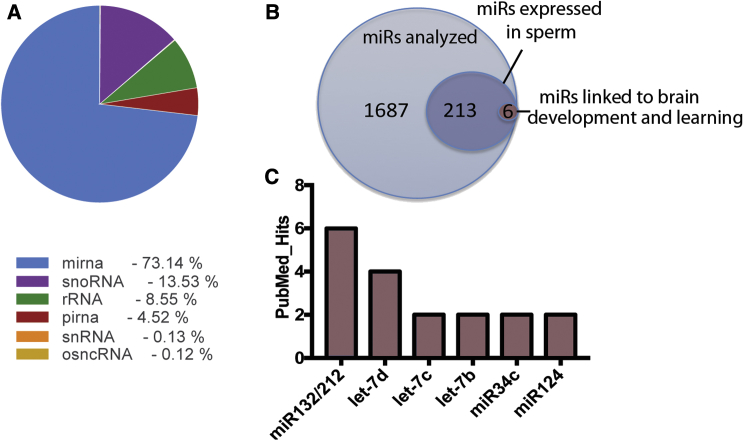
Small RNA-Seq from Sperm Reveals Candidate miRs Potentially Linked to Intergenerational Transmission of Enhanced LTP after EE (A) Pie chart illustrating the distribution of small RNA species in sperm obtained from naive WT mice. (B) Venn diagram showing that, out of 1,886 miRs analyzed, 219 were expressed in sperm. This list of miRs was then searched for miRs implicated in brain function and development, synaptic plasticity, and learning and memory (PubMed search criteria “brain + learning + microRNA-X (let-X)”; more than 1 hit). This approach revealed 6 candidate miRs. (C) Graph showing the 6 candidate miRNAs and the corresponding number of hits based on the PubMed search. PubMed IDs for the papers linked to miR212/132 are 22845676, 22246100, 19557767, 23520022, 27392631, 20613834; let-7d are 23425148, 21307844, 20557304, 25799420; let-7c are 25962166, 21676127; let-7-b are 21676127, 27539004; miR34c are 26402112, 21946562; miR124 are 24784359, 22837048.

**Figure 3:**
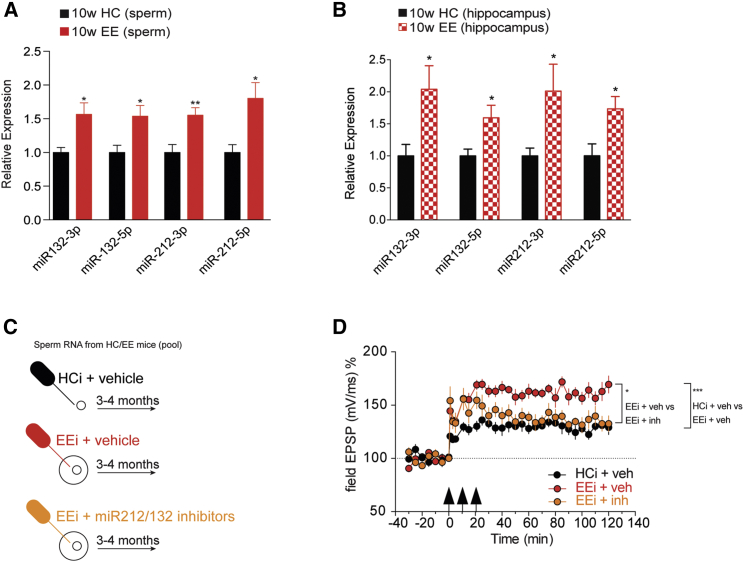
miR212/132 Are Increased in the Brain and Sperm of EE Males and They Are Involved in Intergenerational Inheritance of the Enhanced LTP Phenotype (A) qRT-PCR from sperm of 10-week EE males (n = 7) demonstrates increased expression of miR212/132 when compared to HC males (n = 6; ^∗∗^p < 0.01, ^∗^p < 0.05, t test). (B) qRT-PCR from hippocampus of 10-week EE males demonstrates increased expression of miR212/132 (n = 9/group; ^∗^p < 0.05, t test). (C) Oocyte injection scheme. Sperm RNA from a pool of HC or EE mice was isolated and mixed with scrambled negative control (vehicle) or miR212/132 inhibitors and injected into the cytoplasm of fertilized oocytes. Control (“HCi + vehicle”) oocytes were injected with sperm RNA from HC mice + scrambled negative control. “EEi + vehicle” oocytes were injected with sperm RNA from EE mice + scrambled negative control. “EEi + miRNA212/132 inhibitors” oocytes were injected with RNA from EE mice + miR212/132 inhibitors. The mice born from these injected fertilized oocytes were used for LTP measurements at the age of 3–4 months. (D) LTP is significantly elevated in animals born from EE-sperm-RNA-injected oocytes (“EEi + veh”) compared to HC-sperm-RNA-injected controls (“HCi + veh”), and coinjection with miR-212/132 inhibitors (“EEi + inh”) reverses the LTP enhancement. ^∗∗∗^p < 0.001 repeated-measures ANOVA, main effect “HCi + veh” versus “EEi + veh” (F(1,8) = 52.90). ^∗^p < 0.05 repeated-measures ANOVA, main effect “EEi + veh” versus “EEi + inh” (F(1,9) = 6.099). n = 5 (HCi + veh); 5 (EEi + veh); 6 (EEi + inh). Error bars indicate SEM.

Since the mothers were never exposed to EE, which might have affected maternal care, the enhanced synaptic plasticity observed in the offspring of fathers that underwent EE training must be associated with changes in the father’s gametes. Previous reports linked sperm RNA to transgenerational inheritance and observed, for example, that individual miRs were altered in sperm of mice that passed an acquired anxiety phenotype to the next generation (Gapp et al., 2014). Apart from this, there is additional evidence that manipulating miRs levels in gametes can alter the offspring’s phenotype (Rassoulzadegan et al., 2006, Rodgers et al., 2015). At the same time, miRs are known to play key roles in promoting synaptic plasticity (Fischer, 2014, Schratt, 2009). We therefore hypothesized that miRs might play a role in the intergenerational transmission of EE-induced LTP enhancement. First, we measured the sperm microRNAome detectable in mice used in our experimental system. Out of 1,886 miRs present in the mouse genome, 219 were identified in sperm (Figures 2A and 2B). We subjected these 219 miRs detected in sperm to a PubMed search according to the following criteria: (1) expression in the brain, (2) having been linked to brain plasticity and memory function, and (3) having a documented role in brain development since this might be a possible route of action by which miRs present in gametes could affect brain function in the offspring (Figure 2B). Using these criteria, we identified 6 miRs that showed more than 1 PubMed hit, namely, miR212/132, let-7d, let-7c, let-7b, miR34c, and miR124. MiR212/132 was the top hit according to our search criteria (Figures 2B and 2C). These miRs are co-expressed from the same locus, have been shown to affect synaptic function and learning behavior in mice, and play a role in brain development (Clovis et al., 2012, Hansen et al., 2016, Hernandez-Rapp et al., 2015, Remenyi et al., 2013, Tognini and Pizzorusso, 2012). Therefore, we assayed the expression of the miR212/132 cluster in mice upon EE training. We found that miR132 and miR212 were upregulated both in sperm and hippocampus of mice that were exposed to EE for 10 weeks (Figures 3A and 3B). Of note, a shorter duration of EE (2 weeks) was not sufficient to induce the upregulation of these miRNAs in sperm (Figure S2A), while increased levels were observed in the hippocampus (Figure S2B). Furthermore, none of the other miRs we had identified as candidates for the observed intergenerational phenotypes (let-7b, let-7c, let-7d, miR34c, and miR124; see Figure 2), nor randomly selected miRs exhibited altered expression after 10 weeks of EE (Figures S2C and S2D). These data argue against a general increase of sperm miRs in response to EE and led us to hypothesize that miR212/132 might play a role in the intergenerational transmission of the EE phenotype. To test this possibility, we first injected RNA from sperm of HC or EE mice into fertilized oocytes and examined LTP in the corresponding offspring once they were adult. RNA in both groups was co-injected with scrambled RNA allowing us to include a third group in which we injected into oocytes sperm RNA from EE mice along with miR212/132 inhibitor, which we had previously validated for its inhibitory action (Figure S3).

We observed that the offspring born to oocytes injected with RNA from 10-week EE mice exhibited enhanced LTP, which was reversed to control (HC) levels if miR212/132 inhibitors were co-administered (Figures 3C and 3D). These data demonstrate that EE in adult males enhances hippocampal synaptic plasticity in their offspring and that this effect is mediated through sperm RNA causally involving miR212/132.

**Figure 4:**
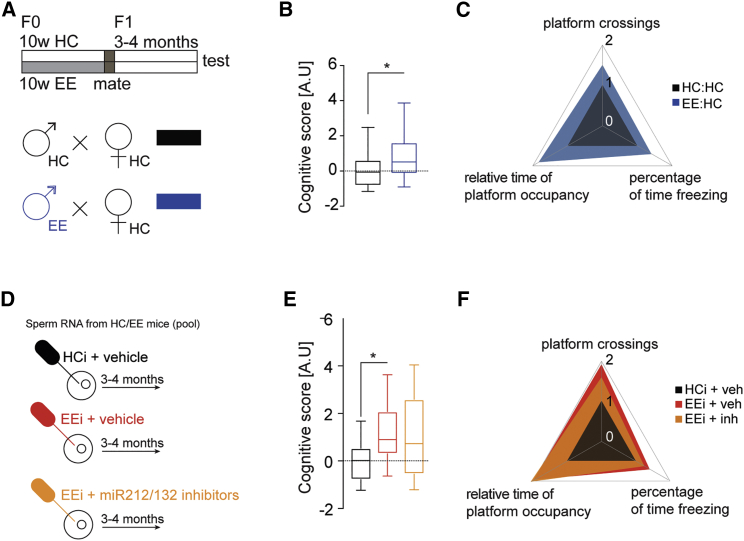
Mice Born to EE Fathers Have a Mild but Significant Cognitive Advantage (A) Breeding scheme. (B) Mice born to EE fathers have a significantly bigger cognitive score. Significance for the F1 generation was calculated using linear mixed models to account for batch and litter effects (see Experimental Procedures). ^∗^p < 0.05 (t-value = 2.80, df = 10). HC:HC: n = 29, N = 6; EE:HC: n = 32, N = 7 (n represents number of mice, N represents number in litter). (C) Plot illustrating the magnitude of change of each individual parameter that went into the cognitive score calculation. (D) Oocyte injection scheme. The injections were carried out as described in Figure 2. The mice born from these injected fertilized oocytes were then tested in behavioral tasks at the age of 3–4 months. (E) Injection of EE sperm RNA into fertilized oocytes provides a cognitive advantage to the offspring, as reflected in the significant increase in the cognitive score. (F) Plot illustrating the magnitude of change in the different groups of each individual parameter of the cognitive score. Significance for the offspring of oocyte injections was calculated using a two-tailed Student’s t test (see Experimental Procedures). ^∗^p < 0.05. n = 9 (HC sperm RNA + negative control oocyte injections), n = 14 (EE sperm RNA + negative control oocyte injections), n = 18 (EE sperm RNA + miR-212/132 inhibitor oocyte injections). Error bars indicate SEM.

Next, we wondered whether this enhancement of LTP was accompanied by an improvement in cognitive performance. We therefore subjected the offspring of EE fathers and those of HC controls to behavioral testing (Figure 4A). We observed no difference in explorative behavior and basal anxiety among groups (Figure S4). Next, mice were subjected to two hippocampus-dependent behavioral tests, namely, the contextual fear-conditioning (FC) and Morris water maze (MWM) paradigms. To avoid ceiling effects on learning, mice were trained using rather mild protocols. Thus, the electric footshock applied during FC was 0.5 mA, an intensity that allows the detection of memory improvement. Of note, there was no difference between the groups in pain sensation as measured by their reaction to the footshock (Figure S4E). In the MWM, mice were trained for a maximum of 5 days, a protocol that in our hands results in a moderate memory consolidation of the platform location in wild-type (WT) animals, which normally form robust spatial memory only after 8 training days (Figure S5).

First, we tested mice in the contextual FC. We observed that mice born to EE fathers showed elevated freezing behavior when compared to mice born to HC fathers (Figures S6A and S6B, t test, p < 0.05 for HC:HC versus EE:HC group). Similar results were observed in the water maze paradigm for platform crossings and relative time of platform occupancy (Figures S6C and S6D, t test p < 0.05, HC:HC versus EE:HC group). We also employed a more stringent approach using a linear-mixed model for statistical analysis taking into account the effect of the different litters, since for the behavioral experiments we employed more than one mouse/litter. In this analysis, we observed a non-significant trend for memory enhancement in the offspring of EE fathers in the FC and water maze paradigms (percentage of time freezing: t = 1.45, df = 10, p = 0.17; platform crossings: t = 1.79, df = 10, p = 0.1; relative time of platform occupancy: t = 1.99, df = 10, p = 0.07). In sum, these data suggest that the intergenerational effect of EE on memory function is subtler compared to the LTP phenotype. This may in part be due to the fact that the analysis of LTP in hippocampal slice preparation allows for a well-defined and specific readout, while the analysis of an animal’s complex behavior as an estimate of memory function, on the other hand, offers a rather limited dynamic range. Rather than relying on the classical readout of the FC and water maze paradigms, we therefore decided to also calculate an integrated cognitive score based on a principal component analysis (PCA). The parameters included in the integrated cognitive score were (1) relative time of platform occupancy in MWM, (2) number of platform crossings in MWM and (3) percentage of time freezing in FC. The first component of the PCA analysis captures the most variance, and hence we took the scores from the PC1 (principal component 1) as a “cognitive score” that would reflect the overall change in cognitive function. This score revealed a significant difference between animals born to HC and EE fathers (Figures 4B and 4C linear mixed model t-value = 2.80, df = 10, p = 0.018), confirming that there is indeed intergenerational inheritance of a mild, but significant cognitive advantage after EE exposure in the fathers.

Next, we decided to address the question of whether sperm RNA and in particular miR212/132 would play a role in the memory enhancement seen in the offspring born to EE fathers. As described for the LTP experiments (see Figure 3). we isolated RNA from the sperm of EE mice and injected this RNA into fertilized oocytes with scrambled RNA. Oocytes injected with RNA from sperm of HC mice together with scrambled RNA were used as control. Also, here, we included a group where EE sperm RNA was co-injected with miR212/132 inhibitors (Figure 4D).

The born mice were subjected to behavioral testing when they were adult. In accordance with our previous observations on LTP enhancement, the mice that originated from oocytes injected with EE sperm RNA showed improved memory function in the FC (Figures S6E and S6F, p = 0.04, t test) and water maze paradigms (Figures S6G and S6H, platform crossings: p = 0.01, Mann-Whitney test; relative time of platform occupancy: p = 0.04, t test.) when compared to those that originated from oocytes injected with HC sperm RNA. This observation was further corroborated when we analyzed the cognitive score as described above (Figures 4E and 4F), suggesting that similar to the intergenerational inheritance of cognitive enhancement mediated by EE, injection of EE sperm RNA into fertilized oocytes also results in a cognitive benefit. In contrast to its effect on LTP, the miR212/132 cluster appeared to have no effect on the behavioral readout. Mice that were born from oocytes injected with EE sperm RNA and co-injected with miR212/132 inhibitors exhibited a similar, albeit insignificant, trend for memory enhancement (Figures 4E and S6F–S6H; percentage time freezing: p = 0.28, Mann-Whitney test; platform crossings: p = 0.19, Mann-Whitney test; relative time of platform occupancy: p = 0.08, t test). These findings suggest that the intergenerational effect of EE on LTP and memory enhancement critically depends on sperm RNA and that the LTP effect is mediated via altered levels of miR212/132. In contrast, miR212/132 levels cannot explain the enhanced memory function indicating that additional mechanisms contribute to the intergenerational enhancement of learning behavior in response to EE. Finally, miR212/132 are not upregulated in the offspring of EE fathers (Figure S8), which suggests that the mechanisms underlying EE-mediated enhanced synaptic plasticity and cognition in the F0 and F1 generation are likely to be different. This interpretation could also explain why the effect of EE in our study is inter- and not transgenerational.

## Discussion

Our data demonstrate that EE during adulthood mediates the enhancement of hippocampal LTP in the adult offspring. This is not only the first confirmation, but also an important extension to the original observation by Arai et al. (2009), who showed that EE in juvenile mice (2 weeks of age) enhances LTP in their offspring. Our observation that this phenomenon occurs even when EE is initiated at a time point at which brain development is complete has important implications, suggesting that exercise before conception could provide a brain plasticity benefit to the offspring. Interestingly, Arai et al. observed that EE-mediated intergenerational enhancement of LTP is transmitted through the mothers but not the fathers. This can be explained by the fact that the authors subjected mice to EE before sexual maturity (i.e., at 14 days of age) for 2 weeks, when female gametes are already present. In contrast sperm production begins only at sexual maturity when mice are 6–8 weeks of age. These facts can plausibly explain why 2 weeks of EE in males starting at the age of 14 days failed to elicit intergenerational transmission. Thus, in our study EE was initiated when mice were 10 weeks of age and thus sexually mature. Moreover, we provide evidence that, in addition to enhanced LTP, offspring of EE fathers exhibit a mild but significant cognitive advantage when compared to offspring of HC fathers. When compared to the LTP effect, the memory enhancement was, however, moderate. One explanation could be that the analysis of Schaffer collateral CA1 LTP in hippocampal slice preparation is much more sensitive and thus better suited to detect increased or decreased plasticity compared to behavioral assays that offer a limited dynamic range for the detection of changes. In line with this, calculation of a combined cognitive score on the basis of two hippocampal memory tests using PCA showed that offspring born to EE fathers or from oocytes treated with RNA isolated from sperm of EE mice exhibit a significant cognitive benefit. It also has to be considered that in our study we analyzed memory function in healthy WT mice. It will thus be interesting to see whether EE training in fathers would provide a benefit in synaptic plasticity and memory function to offspring in a situation when brain plasticity is challenged as it is for example the case during aging or in neurodegenerative conditions. In support of this view, recent findings show that EE partially ameliorates the detrimental transgenerational effects observed in offspring born to parents that were exposed to stressful experiences (Gapp et al., 2016). Similarly, it will be interesting to see whether increased brain plasticity is observed in offspring when the mother undergoes EE training. In this study, we did not address this issue and rather focused on EE fathers since studying the corresponding gametes for subsequent mechanistic analysis is more suited for an initial study. It is, moreover, important to notice that the male mice used for mating were removed from the cage upon conception and thus never came to contact with the offspring. It is thus highly unlikely that the described phenotypes in offspring are due to differences in maternal care. Further support for this view stems from the observation that mice born from fertilized oocytes that were injected with sperm RNA of EE mice recapitulate the phenotype observed in the offspring of EE fathers. Moreover, offspring born to enriched parents and raised by a HC housed dam also display enhanced memory formation (Figure S7). It will be important for future research to test whether EE training of adult female mice will also transmit a synaptic plasticity and cognitive benefit to offspring and to elucidate the underlying mechanisms. Especially interesting will be to see whether EE induces upregulation of miR212/132 in oocytes and whether this or other processes play a role in the intergenerational transmission of EE-mediated enhanced synaptic plasticity and cognition. Such experiments have to take into account a number of additional issues such as the fact that most protocols to collect oocytes include a superovulation regime that could potentially confound oocyte plasticity such as miR expression.

Our data show that the intergenerational inheritance of an EE-induced phenotype involves changes in sperm RNA. This finding is in line with previous studies that investigated transgenerational effects in mutant mice (Rassoulzadegan et al., 2006), in mice exposed to specific stressors leading to altered glucocorticoid signaling (Rodgers et al., 2013, Rodgers et al., 2015), anxiety behavior (Gapp et al., 2014), or diet-induced obesity (Grandjean et al., 2015). An important difference to most of these studies is that we observe an inter- but not a transgenerational effect; i.e., an EE-induced brain plasticity benefit was detectable in the F1 but not in the F2 generation. From an evolutionary point of view, our finding could make sense, since nature offers to the organism a physiological system that allows for non-genetic inheritance of a cognitive benefit in situations of demand but makes sure that these phenotypes do not persist when the environmental settings change again. Taking into account that “too much” plasticity and the resulting aberrant neuronal activity have been linked to neurodegenerative diseases (Fischer et al., 2005, Mesulam, 1999, Palop et al., 2006), it appears logical that EE-mediated LTP enhancement is restricted only to the next generation. Moreover, the EE-mediated intergenerational changes in brain function appear to be regulated at multiple levels. Our data clearly show that one of these mechanisms is related to sperm RNA. In this context, it is interesting to note that the EE-mediated intergenerational inheritance of LTP enhancement was linked to the action of miR212/132. These data are also in line with an earlier study demonstrating that oocyte injection of a mixture of eight miRs could recapitulate the transgenerational effects observed in response to chronic paternal stress (Rodgers et al., 2015). However, inhibition of miR212/132 in fertilized oocytes injected with sperm RNA from EE mice did not occlude the inheritance of improved memory function assayed in the behavioral paradigms. These data clearly indicate that there must be additional miRs and other RNA-dependent mechanisms of similar importance. Moreover, we cannot exclude that non-RNA mediated processes also contribute to this phenotype. In fact, altered DNA methylation (Franklin et al., 2010, Gapp et al., 2016) and histone modifications or the replacement of canonical histones with non-canonical variants (Siklenka et al., 2015, Zeybel et al., 2012) have been implicated in intergenerational inheritance. These findings also suggest that the intergenerational effects of EE-mediated LTP improvement can be dissociated from the improvement of learning behavior at the molecular level, which is in line with the view that, although LTP has been considered a molecular correlate of memory function, it cannot fully explain all aspects of memory consolidation (Titley et al., 2017).

An experiment that could have provided additional evidence for the involvement of miR212/132 in the intergenerational transmission of enhanced LTP would be to inject these miRNAs specifically into fertilized oocytes and perform the tests on the resulting progeny. However, we decided against this “gain-of-function approach.” Convincing data show that downregulation of miR212/132 leads to impairment of synaptic plasticity and learning and memory (Hansen et al., 2010, Hansen et al., 2013, Scott et al., 2012). However, while memory enhancement was observed if miR132 overexpression is 1.5- to 2-fold (Hansen et al., 2013), which is similar to the increase seen in our study in response to EE, transgenic mice overexpressing miR132 more than 3-fold exhibit learning impairment (Hansen et al., 2010, Hansen et al., 2013, Scott et al., 2012). Importantly, the learning impairment caused by supra-physiological upregulation of miR132 in these transgenic mice could be turned into learning enhancement by mitigating miR132 overexpression to a level representing a 1.5- to 2-fold increase (Hansen et al., 2013). These data suggest that moderate increase in miR212/132 facilitates, whereas excess increase in its expression impairs learning and memory, a finding that is not uncommon for molecules implicated with memory function (Fischer et al., 2005). Thus, we suggest that in case of miR212/132 our “loss-of-function” experiment is the most reliable experimental approach to support a role of miR212/132 in intergenerational inheritance.

The precise mechanisms by which sperm RNA transmit EE-induced intergenerational enhancement of brain function to adult offspring remain to be identified. One possibility is that sperm miRs alter gene expression during embryonic development thereby causing subtle changes in brain plasticity. This hypothesis appears worthwhile to be addressed in future studies, since even small changes in brain development can affect synaptic function in the adult organism and are believed to contribute, for example, to neuropsychiatric diseases (Faa et al., 2016, Mahmoudi and Cairns, 2017). Of note, miR212/132 play a role in neurodevelopment as well as the adult brain and have been linked to neurodevelopmental brain diseases such as schizophrenia (Clovis et al., 2012, Miller et al., 2012). These data may also help to understand why EE-induced transmission of LTP enhancement is only observed in the F1 but not in the F2 generation. We speculate that the mechanisms underlying enhanced brain plasticity in the parents and the offspring are different. In the case of the offspring, these mechanisms may be linked to neurodevelopmental processes, a hypothesis to be tested in future studies. In support of this view, we observed that hippocampal levels of miR212/132 increase in fathers exposed to EE (F0 generation; see Figure 3), a finding that is in line with previous data linking moderately increased miR212/132 levels to memory enhancement (Hansen et al., 2016, Hernandez-Rapp et al., 2015, Scott et al., 2012), but not in their adult offspring (F1 generation; see Figure S8). An equally interesting question relates to the mechanisms that lead to increased miR212/132 levels in sperm. Changes in DNA-methylation or histone-modifications during spermatogenesis are likely mechanisms (Gapp et al., 2014, Zeybel et al., 2012). However, other mechanisms should also be considered. For example, our data show that miR212/132 are already increased after 2 weeks of EE in the hippocampus, but only after 10 weeks of EE in sperm. These data could indicate that expression of miR212/132 in the brain is more responsive to external stimuli than that in sperm. Alternatively, there is evidence that brain-derived exosomes, which are known to carry also miRs, have been detected in the circulation (Shi et al., 2014), and thus it is not entirely impossible that increased levels of miR212/132 in sperm originate from increased miR212/132 expression in other tissues such as the brain. Clearly, more research is required to address these questions.

In conclusion, the idea that EE training in adulthood provides a cognitive benefit not only to the individual undergoing this procedure, but also to its offspring is fascinating. Whether these findings are translatable to humans needs to be determined. Nevertheless, the accumulating evidence that sperm RNA content encodes information about environmentally induced phenotypic traits is an issue that not only needs to be considered in reproductive medicine, but may also offer the chance to discover biomarkers for complex diseases.

## Experimental Procedures

### Animals

All procedures were performed according to protocols approved by the Lower Saxony State Office for Consumer Protection and Food Safety. WT C57B/6J animals were ordered from Janvier labs at 9 weeks of age and were allowed to habituate to the animal facility and the group for 1 week. Afterward, they were housed either in standard HC or under EE conditions as described below. Food and water were provided *ad libitum*. Animals were kept on a 12-hr/12-hr-light/dark cycle. For all experiments, including mating, adult mice were used, which refers to an age of 3–4 months.

### EE

For EE, mice were kept in groups of 4–5 in large cages and provided with toys (in the form of tunnels, housing, and differently shaped objects, 8 per cage) and running wheels (2 per cage). 2 toys were replaced daily with novel ones, and the rest was rearranged inside the cage. Cages were changed weekly altogether. Mice were put in the EE at 10 weeks of age and kept there for 10 weeks. HC animals were also kept in groups of 4–5 in large cages in the absence of objects and running wheels but subjected to the same cleaning schedule.

### Luciferase Assay

For luciferase assays, the complementary seed sequence for miR-212/132 was cloned into pmirGLO (Promega) into PmeI/XbaI sites by oligo hybridization (see below). The constructs were cotransfected into HEK293 cells with miR-212/132 mimics (5 nM, QIAGEN) or miR-212/132 inhibitors (5 nM for 3p arms, 50 nM for 5p arms, Exiqon) using lipofectamine according to the manufacturer’s instructions. Luciferase measurements were taken 48 hr in a Tecan plate reader using Promega’s Dual-Luciferase Reporter Assay System following manufacturer’s instructions. The Firefly/Renilla ratio was calculated and then normalized to control conditions.

### Oligonucleotide Sequences Used for Cloning

The following oligonucleotides were used for cloning the miRNA target sequences into pmirGLO:

miR-132-3p F: AAACTAGCGGCCGCTAGTCGACCATGGCTGTAGACTGTTA
miR-132-3p R: CTAGATAACAGTCTACAGCCATGGTCGACTAGCGGCCGCTAGTTT
miR-132-5p F: AAACTAGCGGCCGCTAGTGTAACAATCGAAAGCCACGGTTT
miR-132-5p R: CTAGAAACCGTGGCTTTCGATTGTTACACTAGCGGCCGCTAGTTT
miR-212-3p F: AAACTAGCGGCCGCTAGTTGGCCGTGACTGGAGACTGTTAT
miR-212-3p R: CTAGATAACAGTCTCCAGTCACGGCCAACTAGCGGCCGCTAGTTT
miR-212-5p F: AAACTAGCGGCCGCTAGTAGTAAGCAGTCTAGAGCCAAGGT

### Sperm Collection and RNA Isolation

Animals were sacrificed by cervical dislocation. Sperm was isolated by dissecting the epididymis and running a needle through the tube to allow sperm to swim out into 1 mL of pre-warmed PBS. Epidydimal tissue was kept at 37°C for 20 min, and the supernatant was then collected and centrifuged at 8,000 × *g* for 5 min at 4°C. In order to remove contaminating epithelial cells prior to RNA isolation, the pellet was treated with hypotonic buffer (0.1% SDS, 0.5% Triton-X) for 30 min on ice to ensure that endothelial cells from the surrounding tissue were disrupted. The solution was centrifuged once more at 8,000 × *g* for 10 min at 4°C to obtain the final sample. RNA was isolated using Tri-Reagent from Sigma-Aldrich according to manufacturer’s instructions. RNA was treated with DNase I (Thermo Fisher Scientific) and further purified using phenol:chloroform to remove all DNase I components. RNA concentration was determined with the Nanodrop and quality with the Bioanalizer.

### qRT-PCR

cDNA synthesis was done using QIAGEN’s miScript II Reverse Transcription kit II according to manufacturer’s instructions starting from 250 ng of DNase-I-treated RNA. qRT-PCR was performed using QIAGEN’s commercially available miScript Primer Assays (i.e., forward primers) specific for each miRNA combined with miScript Universal Primer and miScript SYBR Green Master Mix. miRNA levels were normalized to RNU6B. 2–5 ng of cDNA was used per reaction. Assays were run in duplicate. For miR212/132, the qRT-PCR was performed for both active and inactive arms. For all other miRNAs, only active arms were analyzed. The ΔΔCt method was used to calculate relative expression.

### RNA Injections into Fertilized Oocytes

Sperm RNA from 4 HC and 4 EE animals was isolated as described above. RNAs were pooled and set at 0.5 ng/μL in 250 μL 0.1 mM EDTA, 5 mM (pH 7.4) based on a previous publication reporting significant effects at this concentration (Gapp et al., 2014). 3- to 4-week-old C57BL/6JRj females were super-ovulated with pregnant male serum gonadotropin (7.5 U, Intervet) and human chorionic gonadotropin (7.5 U, Intervet) and mated with C57BL/6JRj males. Donor females were sacrificed by cervical dislocation on the day of plug, and fertilized eggs were collected. RNA was injected into the cytoplasm of zygotes at the pronuclear stage using an Eppendorf Femtojet and Femtotip II capillaries with constant flow under visual control on an inverted microscope using a 40× air objective and differential interference contrast (DIC) optics. Injected zygotes were transferred into the oviduct of pseudo-pregnant NMRI fosters bilaterally. The offspring generated from these injections was allowed to grow in a standard cage until 3 month of age before behavioral testing.

### Behavioral Experiments

Animals that went into behavioral experiments were sexually naive and were single-caged 1 week prior to the start of the procedures. For contextual FC, animals were put in a FC box (Med Associates) for 3 min, after which they received a 2-s, 0.5-mA footshock. They were left in the box for 10 s and then removed and put back into their original cage. 24 hr later, animals were reintroduced into the conditioning box, and freezing behavior was recorded for the 3-min period. Freezing was considered when animals remained immobile (except for respiratory movements) for at least 2 s. Note that this FC paradigm represents a rather mild training therefore allowing the detection of memory enhancement, which is in line with HC mice showing relatively low freezing levels. For MWM, animals were trained in a round pool filled with opaque water where the escape platform was placed approximately 1 cm below water level. They were trained in 4 consecutive 1-min trials per day with randomized entry points. If the animals did not reach the platform within 1 min, they were gently guided to it. Animals were allowed to stay on the platform for 15 s after each trial. The time needed to reach the platform (escape latency) was automatically recorded via a top-installed camera (TSE Systems, Bad Homburg, Germany) and registered with the TSE Systems software VideoMot. For the probe test, the platform was removed and animals were introduced in the position opposite of the original platform location and left to navigate the pool for 1 min. The percentage of time spent in the specific region where the platform used to be during training (time of platform occupancy) was recorded with the TSE Systems software. The experimenters were blind to the animals’ experimental condition.

### Breeding Scheme for Intergenerational Analysis

10-week-old male mice acquired from Janvier were subjected to 10 weeks of EE according to the protocol described above. HC and EE male mice were bred with females that had remained HC throughout this time. After mating, the fathers were immediately removed from the cage so that they did not have any contact with their offspring and did not impact upon their rearing. Offspring that originated from these pairings were housed in standard HCs and subjected to LTP measurements or behavioral experiments at 3–4 months of age.

### LTP Recordings

Acute hippocampal slices were prepared from 3- to 4-month-old mice. Animals were anesthetized with isoflourane and decapitated. Brain was removed from the skull and hippocampus was dissected. Transversal hippocampal slices (400 μm thick) were obtained using a tissue chopper (Stoelting). Slices were collected in ice-cold artificial cerebrospinal fluid (ACSF) (124 mM NaCl, 4.9 mM KCl, 1.2 mM KH_2_PO_4_, 2.0 mM MgSO_4_, 2.0 mM CaCl_2_, 24.6 mM NaHCO_3_, 10.0 mM D-glucose; saturated with 95% O_2_ and 5% CO_2_ [pH 7.4] and 305 mOsm). Slices were incubated in an interface chamber at 32C and high oxygen tension was maintained by bubbling with 95% O_2_ and 5% CO_2_ (30 L/hr). Slices were allowed to recover for 3 hr after preparation. Then monopolar platinum-iridium electrodes (13303, MicroProbes) used for both recording and stimulating were positioned in the CA1 region. The field excitatory postsynaptic potential (fEPSP) slope was recorded with a Model 1700 differential AC amplifier (A-M Systems) and Power 1401 analog-to-digital converter (Cambridge Electronic Design), and monitored online with custom-made software, PWIN (IFN Magdeburg). The test stimulation strength was determined for each input as the current needed to elicit a field EPSP of 40% maximal slope. Baseline recording began at least 3.30 hr after slice preparation, using test stimuli consisting of four biphasic constant current pulses (0.2 Hz, 0.1 ms/polarity, averaged) per time point, every 5 min for a minimum of 30 min. LTP was induced with a strong tetanization protocol consisting of three stimulus trains (100 biphasic constant-current pulses per train at 100 Hz and 0.2 ms/polarity, inter-train interval 10 min). Test stimuli were delivered 1, 3, 5, 11, 15, 21, 25, and 30 min after the first tetanization train and then every 5 min for up to 2 hr. The experimenters were blind to the animals’ experimental condition.

### Small RNA Sequencing

RNA isolated from two independent biological replicates of sperm from WT adult mice was used for small RNA sequencing (RNA-seq) library preparation using Illumina’s TruSeq kit according to manufacturer’s instructions starting from 1 μg total RNA. Library quality was checked with the Bioanalyzer (Agilent). For processing of sequencing data, a customized in-house software pipeline was used. Quality check and demultiplexing were performed using the CASAVA 1.8.2 software (Illumina). We trimmed low-quality ends from reads before adaptor removal having Phred quality score of less than 28 encoded in ASCII + 33. We then trimmed the 3′ adapters and filtered out the reads with the minimum length of 15 nucleotides using cutadapt. We first mapped the reads to a custom-made reference genome created from miR and piwi-interacting RNA (piRNA) sequences. Reads greater than 33 bp were mapped to the reference genome created by other small non-coding RNA. Remaining unmapped reads were then mapped to the mouse genome. We used bowtie (v.1.1.2) for mapping. We allowed no mismatches for the first part of mapping and one mismatch for rest of the mapping. Read count distribution and visualization were generated using in-house Python script with Python version 2.7.10.

### Statistical Analysis

Since several animals from the same litter were analyzed and tests were done at two different time points (i.e., batches) to ensure that the group size was homogeneous and stayed within the same light/dark cycle, we used generalized mixed models to account for such type of random effects. We used the R packages MASS (MASS_7.3-45) and nlme (lme4_1.1-12) to construct and evaluate the generalized linear models. We first constructed a model with treatment and sex as fixed factors and litter nested within batch as random factors. There was no significance for sex main effect or for the interaction between sex and treatment, so we dropped the sex effect for evaluating the main effect of treatment. For the oocyte injection experiments, the sperm RNA comes from a pool of 4 HC/EE mice and the manipulation happens at the level of each individual embryo so we considered animals separately. Other analyses were done with GraphPad Prism and corresponding statistical tests are mentioned in the figure legends. All figures represent mean ± SEM. Exact n (mouse) and N (litter) numbers are also specified in figures legends if applicable.

## Data and Software Availability

The accession number for the data reported in this study is GEO: GSE110183.

## Supplemental Information

Document S1. Figures S1–S8

Document S2. Article plus Supplemental Information
