# Supplementary material for: RNA-Dependent Intergenerational Inheritance of Enhanced Synaptic Plasticity after Environmental Enrichment
Source: Cell Rep. 2018 Apr 10;23(2):546–54. doi: 10.1016/j.celrep.2018.03.059 (PMC5912949; doi:10.1016/j.celrep.2018.03.059)
Supplement: Document S1. Figures S1–S8 [file mmc1.pdf]

Cell Reports, Volume *23*

Supplemental Information

RNA-Dependent Intergenerational Inheritance of Enhanced Synaptic Plasticity after Environmental Enrichment

Eva Benito, Cemil Kerimoglu, Binu Ramachandran, Tonatiuh Pena-Centeno, Gaurav Jain, Roman Manuel Stilling, Md Rezaul Islam, Vincenzo Capece, Qihui Zhou, Dieter Edbauer, Camin Dean, and André Fischer

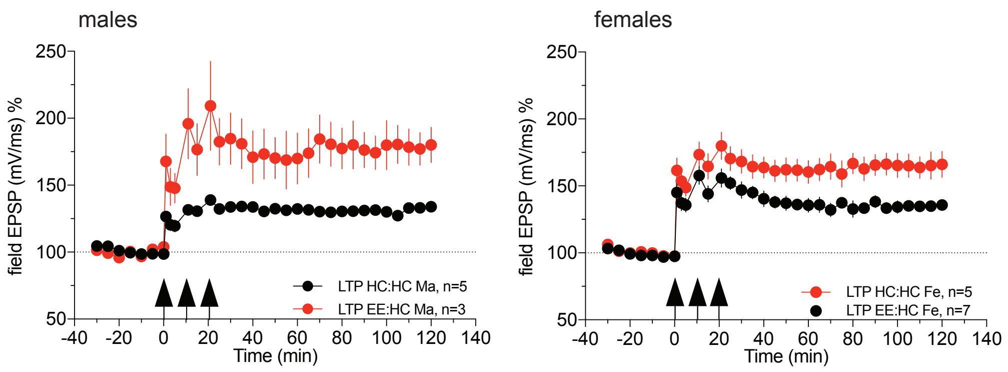

Figure S1. The intergenerational effect of EE on LTP is similar in male and female offspring. Related to Figure 1

LTP recordings shown in Figure 1 split by sex to illustrate that the effect is similar in males and females. Males: * $p < 0.05$ for main effect treatment repeated measures ANOVA (F (1, 6) = 13.09); 5 (HC:HC), n = 3 (EE:HC). Females: * $p < 0.05$ for main effect treatment repeated measures ANOVA (F (1, 10) = 6.293); 5 (10w HC:HC), n = 7 (10w EE:HC). Furthermore, a linear regression model including paternal treatment (HC:HC vs EE:HC), sex (male vs female) and paternal treatment X sex interaction revealed a highly significant effect for paternal treatment (**** $p < 0.0001$). Error bars indicate SEM.

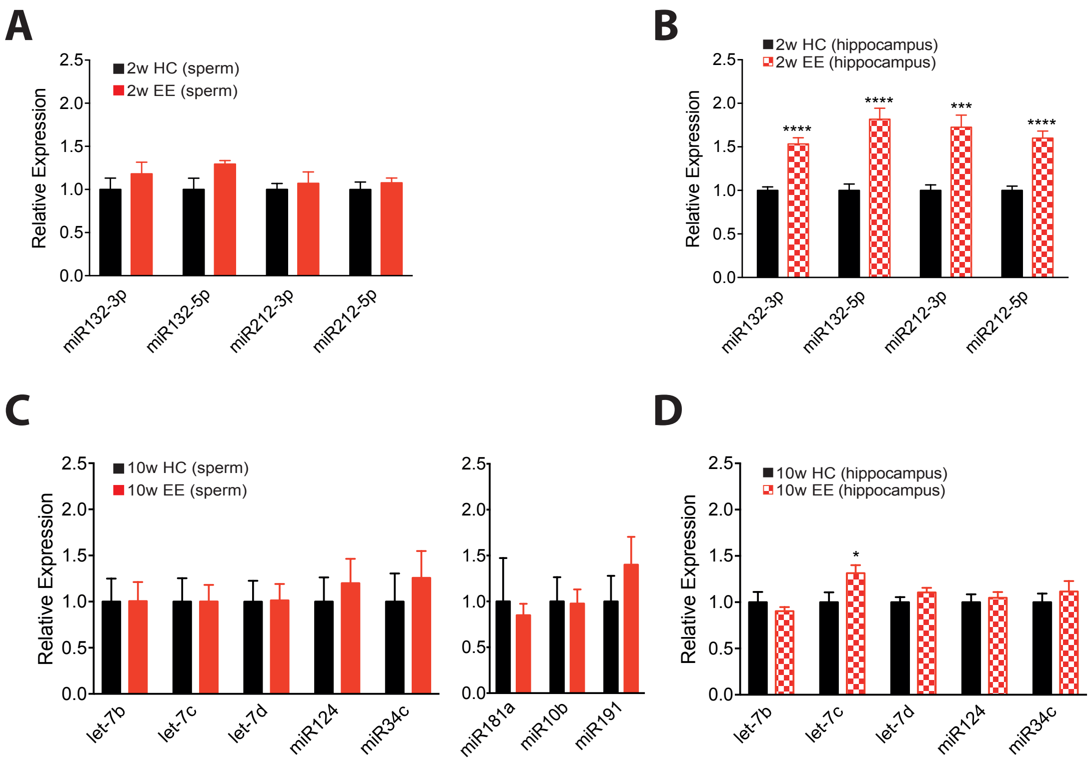

Figure S2 Expression of miR212/132 after EE. Related to Fig. 3 and 4.
A. miR212/132 expression does not change in the sperm after 2 weeks of EE (HC: n = 5; EE: n = 4). **B.** miR212/132 expression in the hippocampus is already increased after 2 weeks of EE (HC: n = 8; EE: n = 7). **C. Left panel.** The other sperm-expressed miRNAs that were previously implicated in learning & memory and brain development according to our Pubmed search (see Figure S2) do not change in their expression in sperm after 10 weeks of EE (HC: n = 11; EE: n = 10). **Right panel.** Randomly selected sperm-expressed miRNAs. Their expression in the sperm also does not change after 10 weeks of EE. **D.** Expression of the other sperm-expressed and learning-related miRNAs in hippocampus. Except an increase in let-7c none of the miRNAs are exhibit changed expression after 10 weeks of EE (n = 9/group). * $p < 0.05$

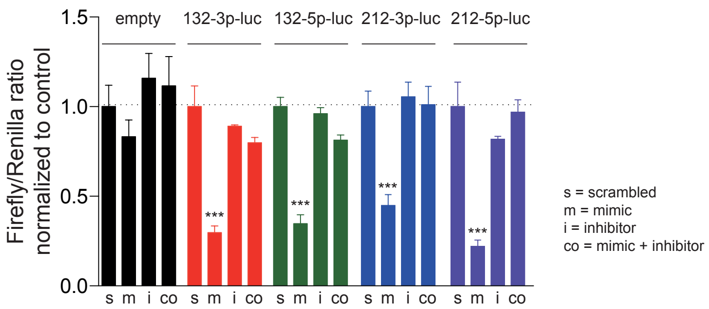

Figure S3. miR-212/132 inhibitors are functional and able to block the effect of miRNA mimics. Related to Fig. 3 and 4.

Luciferase experiment showing that the used miR-212/132 inhibitors are functionally active and block the effect of miR-212/132 mimics on their targets. HEK293 cells were transfected with either the empty pmirGLO reporter or pimrGLO containing the specific miRNA reporter as indicated on top. Mimics and inhibitors were cotransfected and 48h later luciferase measurements were done. b = blank; m = mimic; i = inhibitor; co = cotransfection of mimic and inhibitor. There was no effect of the mimics or inhibitors on the empty pmirGLO plasmid. For all other reporters, mimic expression led to a significant reduction of luciferase signal. The inhibitor alone had no effect on endogenous (human) miRNAs 212/132. However, cotransfection of the reporter and the mimic and inhibitor together led to a significant reinstatement of luciferase signal, indicating that the inhibitor is effectively blocking the action of the mimic on the luciferase target. *** $p < 0.001$, Student's t-test vs. blank control; $n = 9$ for all groups with the empty pmirGLO plasmid; $n = 3$ for all other groups.

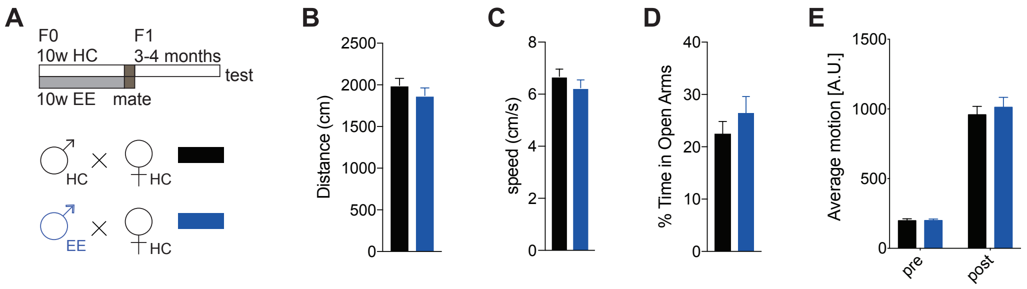

Figure S4. Mice born to EE fathers do not show any motor impairments or change in anxiety levels. Related to Fig. 3 and 4.

A. Breeding scheme. **B.** Distance travelled in the open field is not significantly different between the offspring of HC and EE fathers (t-value = 0.30, Df = 2, p = 0.79). **C.** Speed in the open field is not significantly different between the offspring of HC and EE fathers (t-value = 0.005, Df = 2, p = 0.99). **D.** The percentage of time spent in the open arms of the elevated plus maze is not significantly different between the offspring of HC and EE fathers (t-value = 1.11, Df = 2, p = 0.37). HC-HC: n = 12, N = 2; EE-HC: n = 10, N = 2. **E.** The average motion before the footshock (pre) and in response to (during) the footshock (post) in fear conditioning test is not different between the offspring of HC and EE fathers. HC:HC: n = 31, N = 6; EE:HC: n = 32, N = 7. Please note the increase in average motion before (pre) and during (post) the footshock, indicating that pain sensation is not affected by EE in our experiments.

n: number of animals, N: number of litters.

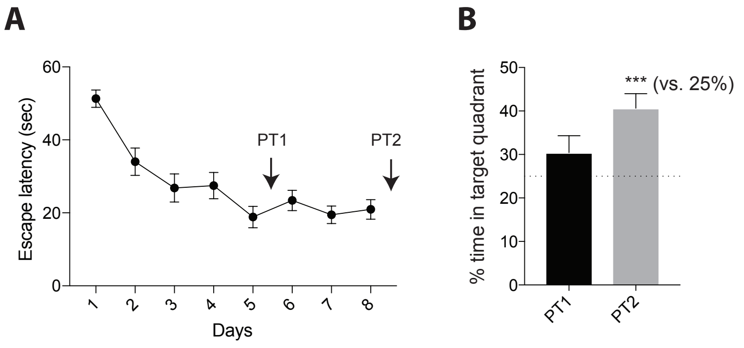

Figure S5. Timeline for memory acquisition in MWM in WT animals in our experimental setting. Related to Fig. 4.

A. Latency curve for WT animals trained daily in the MWM over 8 days. **B.** The percentage of time in the target quadrant is barely above 25% in an early probe test (after 5 days of training) but reaches significant levels after 3 additional days of training (*** $p < 0.001$; one-sample t-test, hypothetical value: 25%).

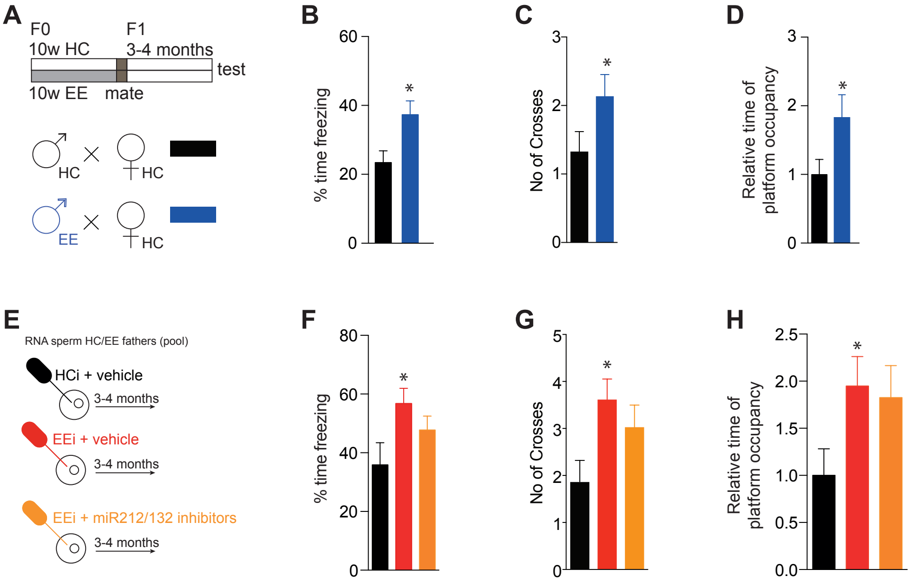

Figure S6. Mice born to EE fathers show a generalized trend towards better cognition in two CA1-dependent tasks. Related top Fig 3 and 4.

A. Mating scheme. **B.** Percentage of time freezing in contextual fear conditioning in offspring (F1). Student's t-test: * $p < 0.05$; generalized linear model: $t = 1.45$, $Df = 10$, $p = 0.17$. HC:HC: $n = 31$, $N = 6$; EE:HC: $n = 32$, $N = 7$. **C.** Number of platform crosses in the Morris water maze probe test in offspring (F1). Student's t-test: * $p < 0.05$; generalized linear model: $t = 1.79$, $Df = 10$, $p = 0.1$. HC:HC: $n = 29$, $N = 6$; EE:HC: $n = 33$, $N = 7$. **D.** Relative time of platform occupancy in the Morris water maze probe test in offspring (F1). Student's t-test: * $p < 0.05$; generalized linear model: $t = 1.99$, $Df = 10$, $p = 0.07$. HC:HC: $n = 29$, $N = 6$; EE:HC: $n = 33$, $N = 7$. **E.** Oocyte injection scheme. See also Fig 2C. **F.** Percentage of time freezing in contextual fear conditioning in mice born from fertilized oocytes. Student's t-test: * $p < 0.05$, HCi: $n = 10$, EEi: $n = 14$, EEi + inh: $n = 18$. **G.** Number of platform crosses in the Morris water maze probe test in mice born from fertilized oocytes. * $p < 0.05$, HCi: $n = 9$, EEi: $n = 14$, EEi + inh: $n = 18$. **H.** Relative time of platform occupancy in the Morris water maze probe test in mice born from fertilized oocytes. * $p < 0.05$, HCi: $n = 9$, EEi: $n = 14$, EEi + inh: $n = 18$. Error bars indicate SEM. n: number of animals, N: number of litters.

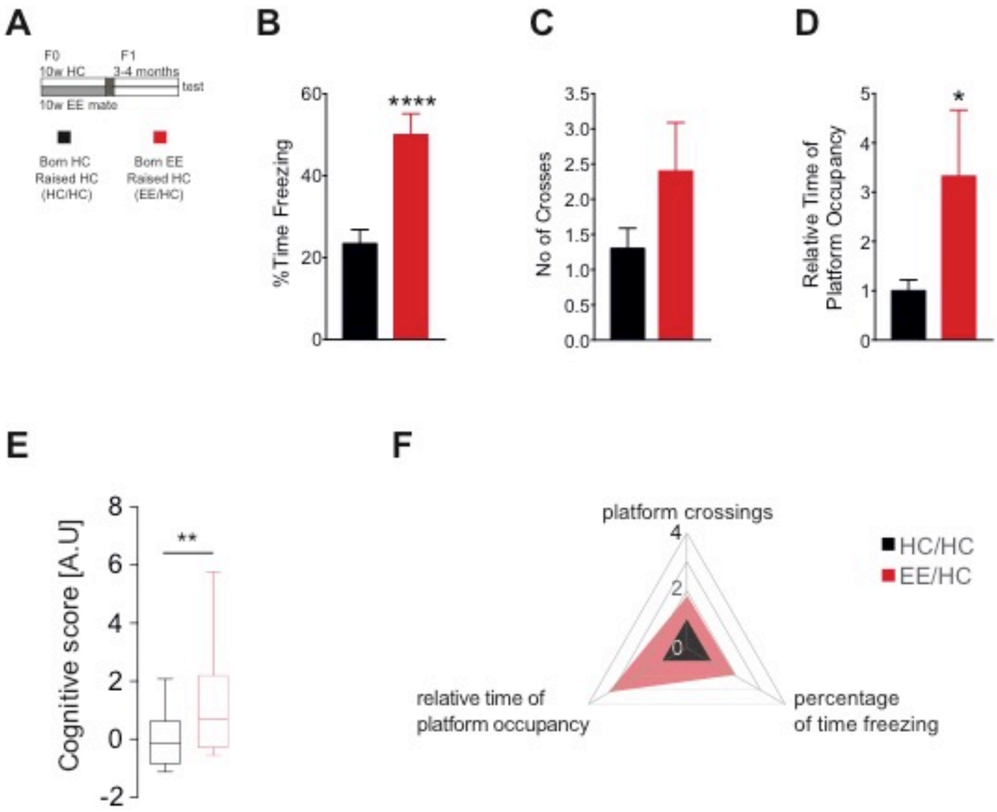

Figure S7: The intergenerational inheritance of acquired cognitive advantage through EE is attributable to germline transmission. Related to Fig. 4.

A. Mating scheme to test the effect of maternal care: after 10 weeks of EE, mice are mated and the offspring are raised by an unrelated HC (home cage) female immediately after birth. **B.** Offspring born to EE parents but raised by an HC female have an improved performance in fear conditioning test compared to controls (HC/HC). Student's t-test: **** $p < 0.0001$; generalized linear model: $p < 0.05$. HC/HC: n = 32, N = 6; EE/HC: n = 15, N = 2. **C.** A similar, albeit insignificant, trend can be observed for platform crosses in the probe test of Morris water maze test. Student's t-test: $p = 0.16$. **D.** Offspring born to EE parents but raised by an HC female have an increased relative time of platform occupancy in the probe test of Morris water maze test compared to controls. Student's t-test: * $p < 0.05$; generalized linear model: $p < 0.1$. HC/HC: n = 30, N = 6; EE/HC: n = 15, N = 2. **E.** Mice born to EE parents and raised by HC female have a significantly bigger cognitive score than controls. Student's test: ** $p < 0.01$. F. Plot illustrating the magnitude of change of each individual parameter that went into the cognitive score calculation. Error bars indicate SEM.

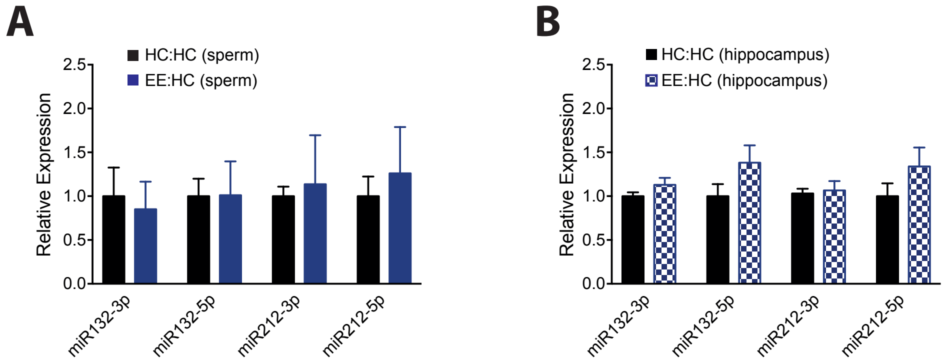

Figure S8. miR212/132 is not significantly changed in the offspring of EE fathers (F1 generation). Related to Fig. 1, 3 and 4.

qPCR analysis for miR212/132 in the F1 generation revealed no significant change in the expression of these microRNAs in sperm (A) (n = 4/group) nor in hippocampus (B) (n = 7/group) when we compared mice of the F1 generation that were born to father that were exposed to 10 weeks of environmental enrichment (EE fathers; EE:HC group) to the corresponding control group that consisted of mice born to fathers that were housed in standard home cages (HC:HC group). Error bars indicate SEM.
